# Supplementary figures and images for: Gene disruption by structural mutations drives selection in US rice breeding over the last century
Source: PLoS Genet. 2021 Mar 18;17(3):e1009389. doi: 10.1371/journal.pgen.1009389 (PMC7971508; doi:10.1371/journal.pgen.1009389)

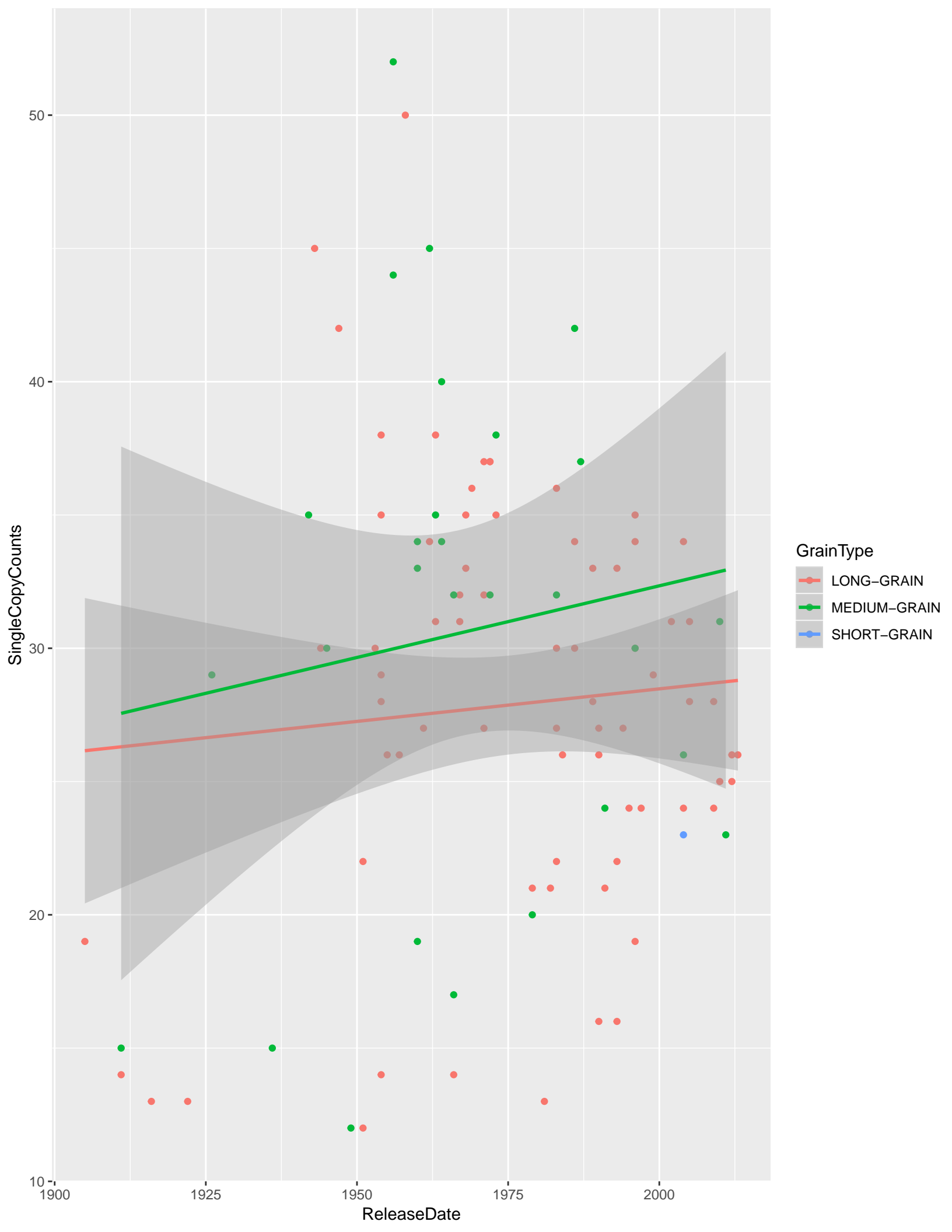

Supplement: S1 Fig — (PDF) [file pgen.1009389.s001.pdf]

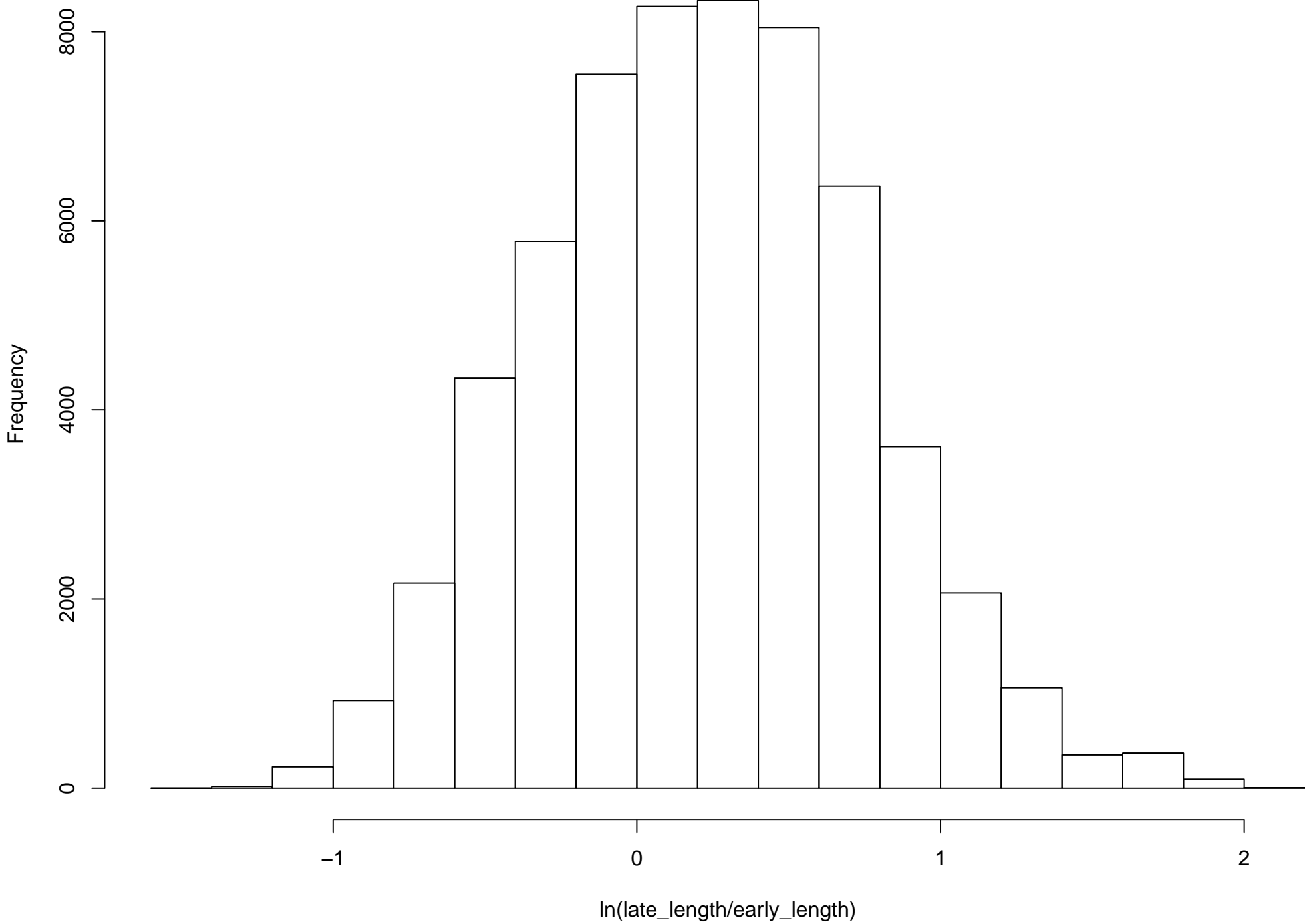

Supplement: S2 Fig — (PDF) [file pgen.1009389.s002.pdf]

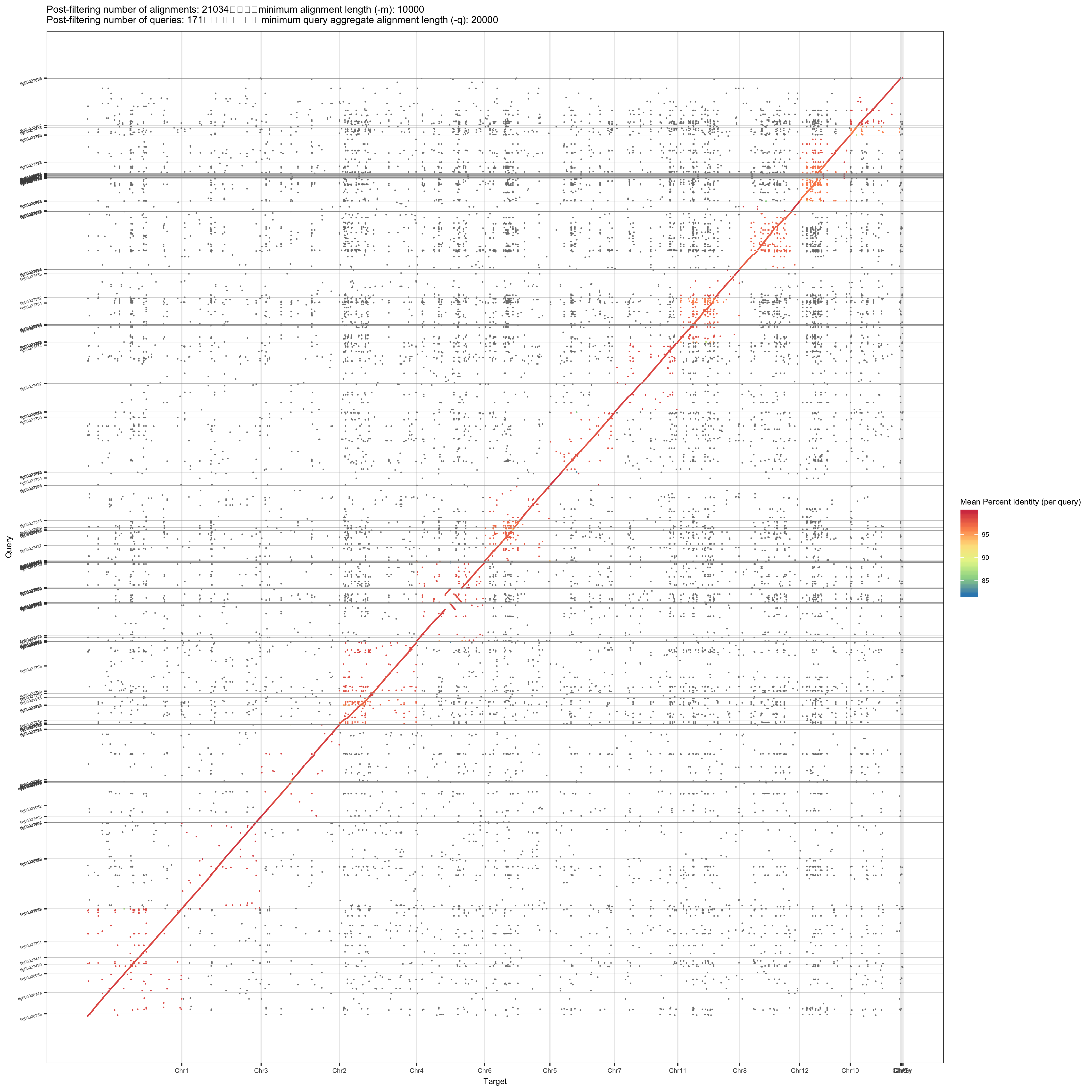

Supplement: S4 Fig — (PNG) [file pgen.1009389.s004.png]

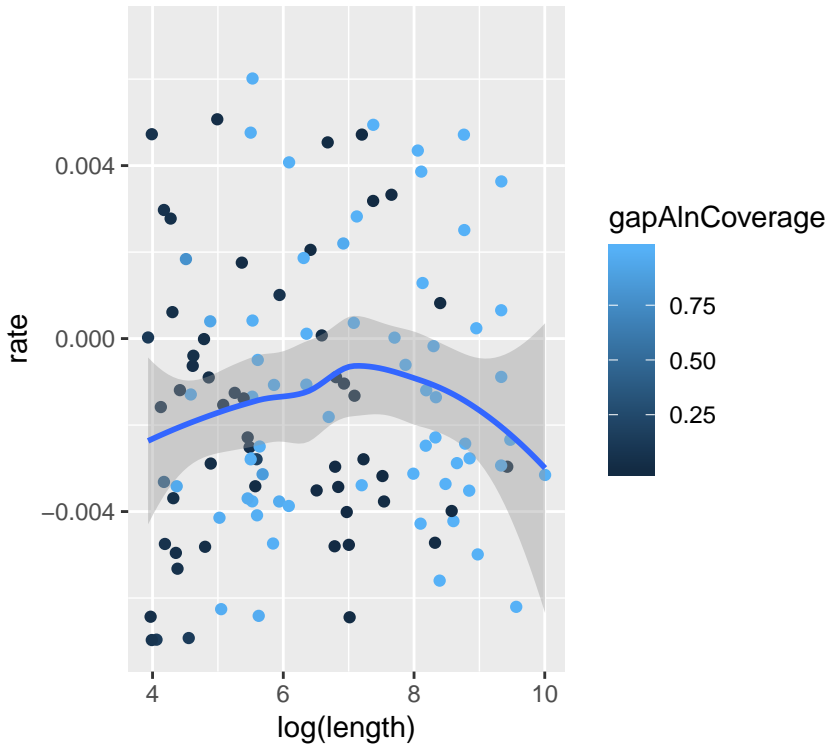

Supplement: S5 Fig — Insertions are light blue; deletions are black. (PDF) [file pgen.1009389.s005.pdf]

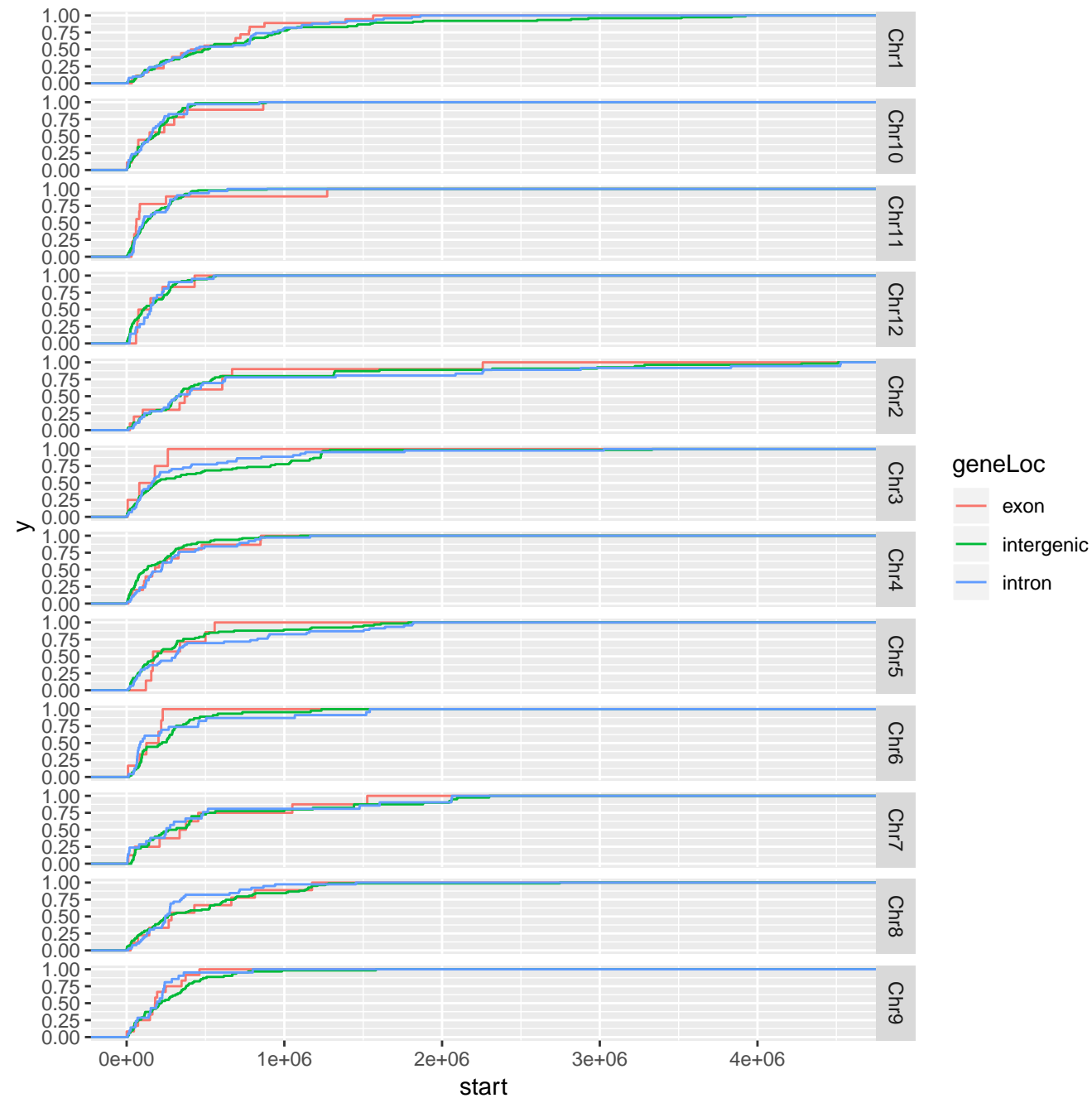

Supplement: S6 Fig — All chromosomes, regardless of length, shared the same x-axis. (PDF) [file pgen.1009389.s006.pdf]

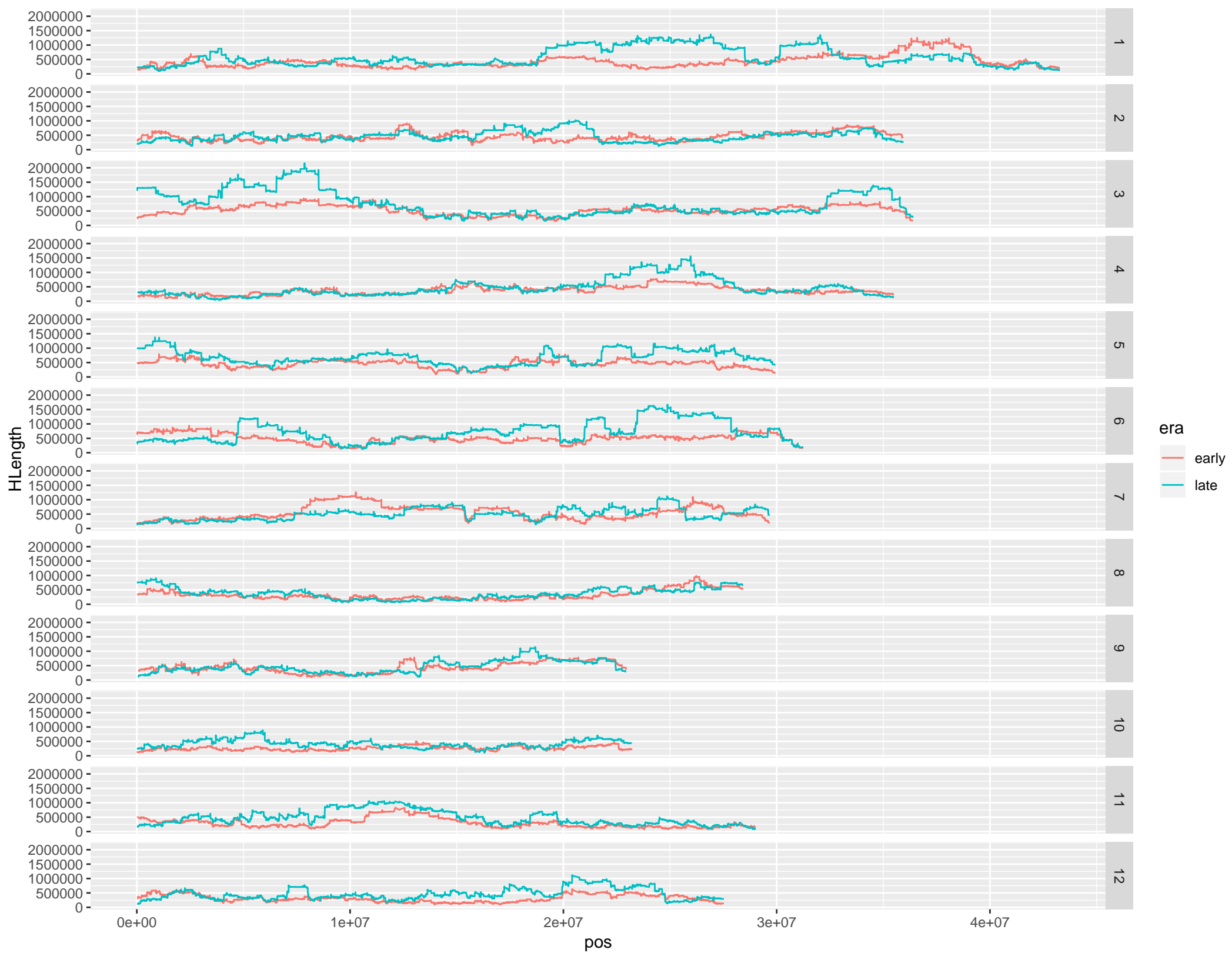

Supplement: S7 Fig — (PDF) [file pgen.1009389.s007.pdf]

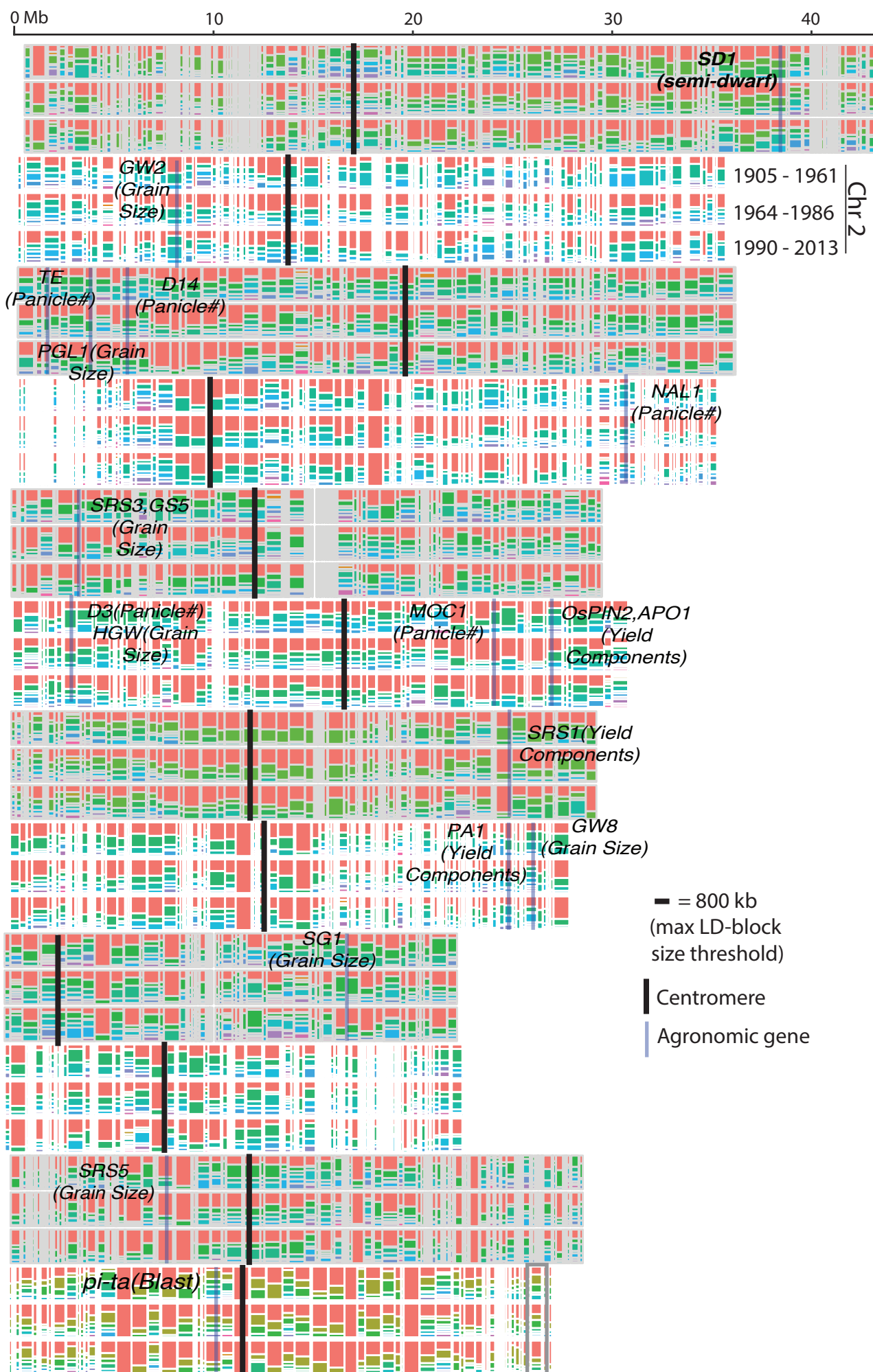

Supplement: S8 Fig — (PDF) [file pgen.1009389.s008.pdf]

Marginal increase in reference (Nipponbare) bias through time

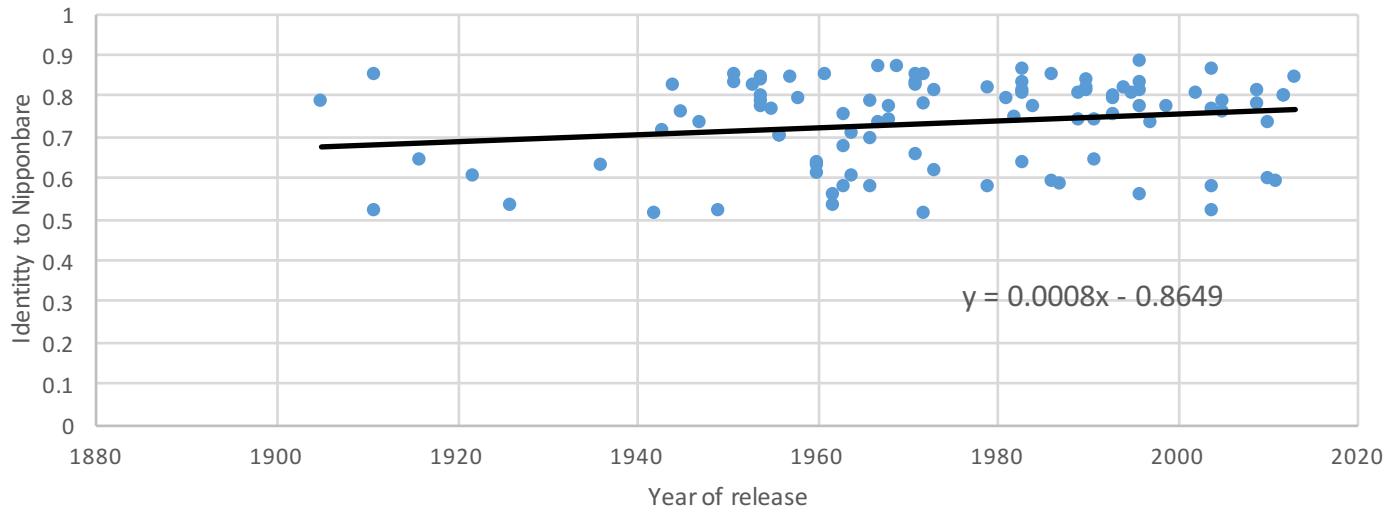

Supplement: S9 Fig — (PDF) [file pgen.1009389.s009.pdf]

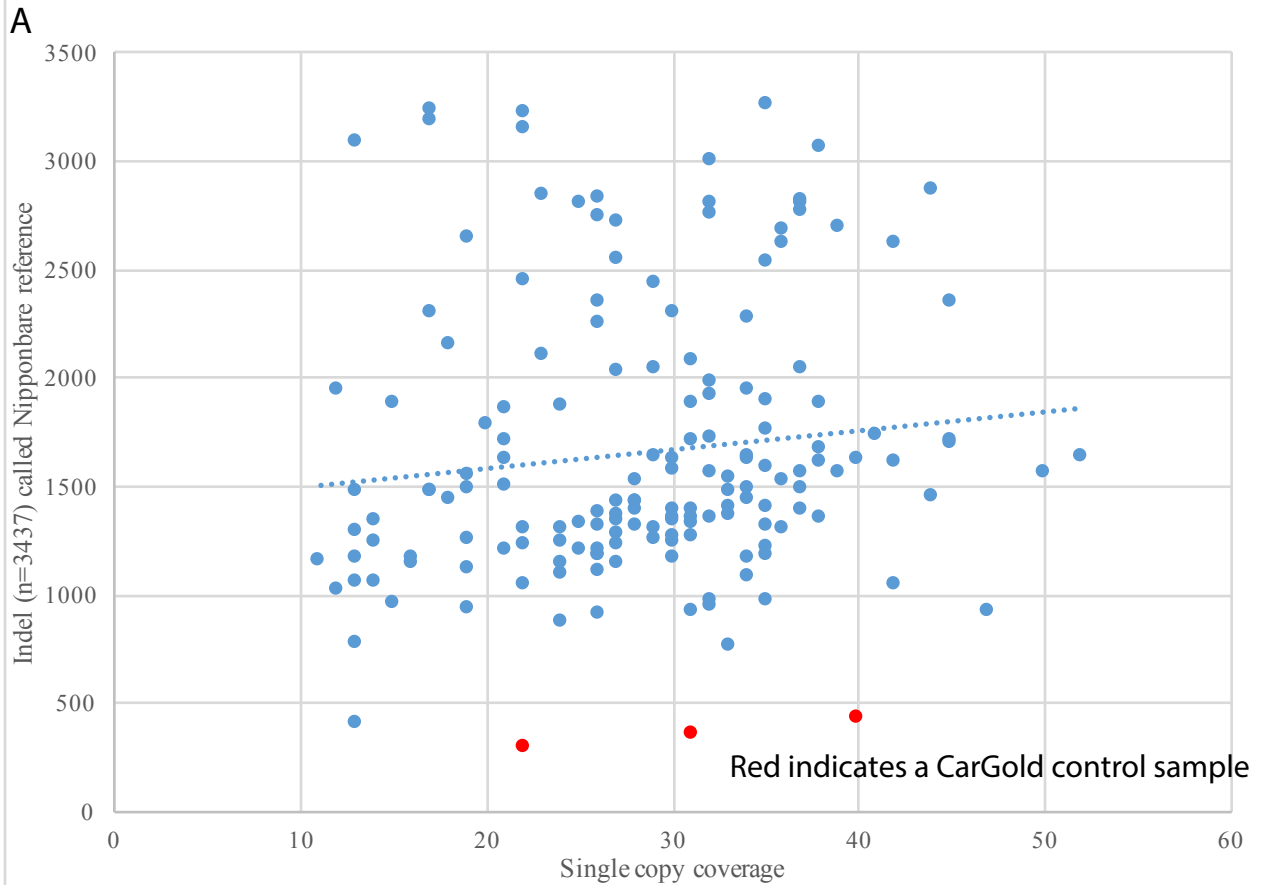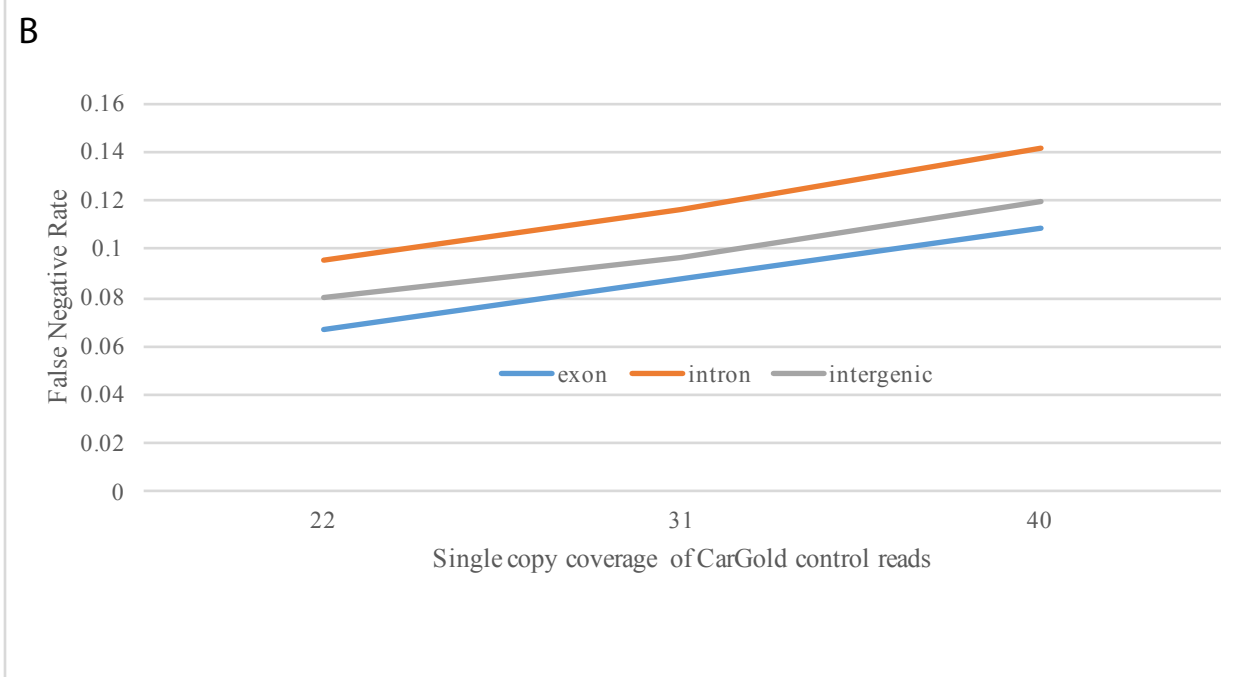

Supplement: S10 Fig — A) Relationship between coverage and genome-wide genotyping as well as downsampled CarGold short-read sets, which are assumed to have zero true Nipponbare alleles based on assemblies. B) Linear relationship between coverage and false negative rate for each variant class. (PDF) [file pgen.1009389.s010.pdf]

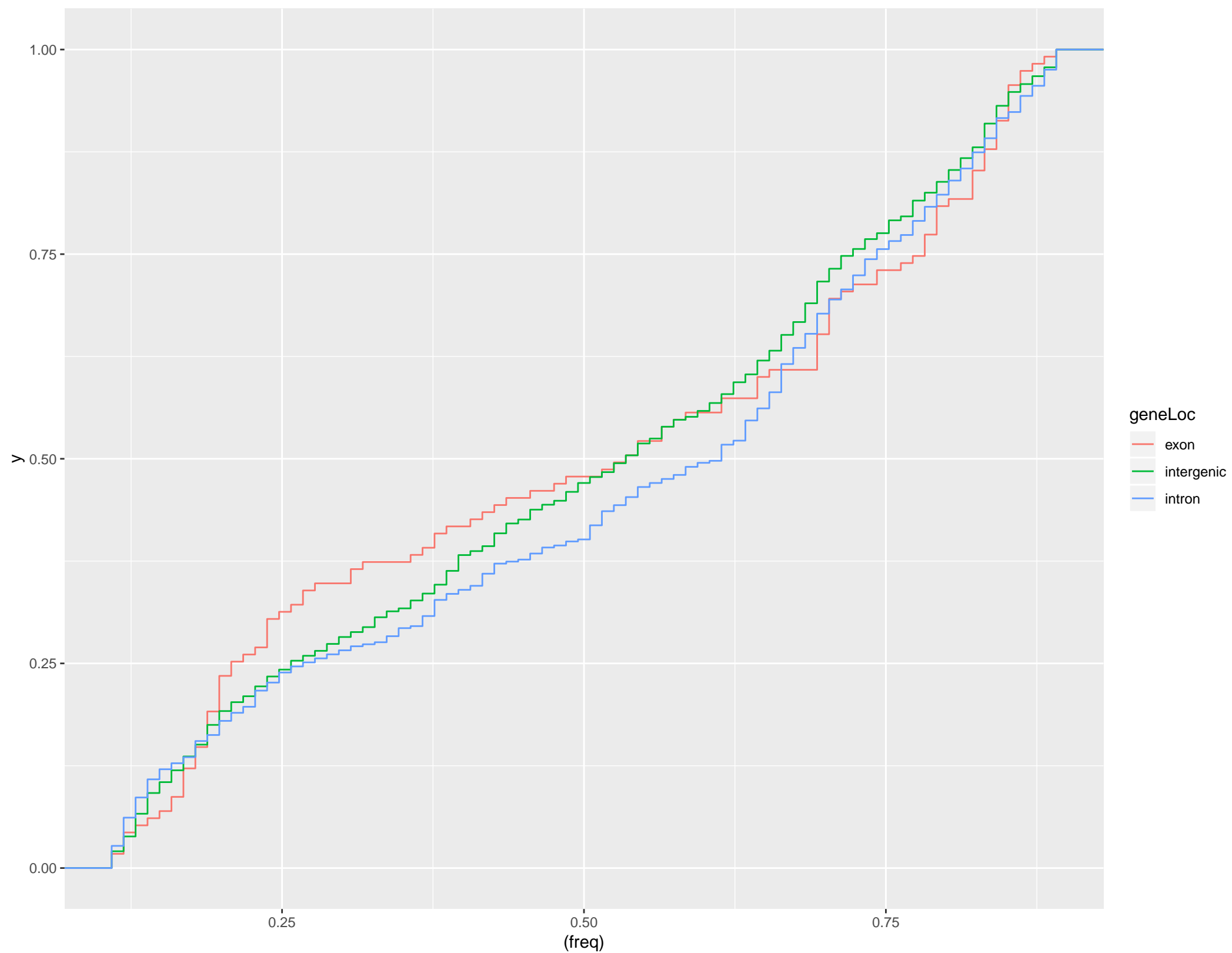

Supplement: S11 Fig — Each line represents the location of the SV relative to a gene. (PDF) [file pgen.1009389.s011.pdf]

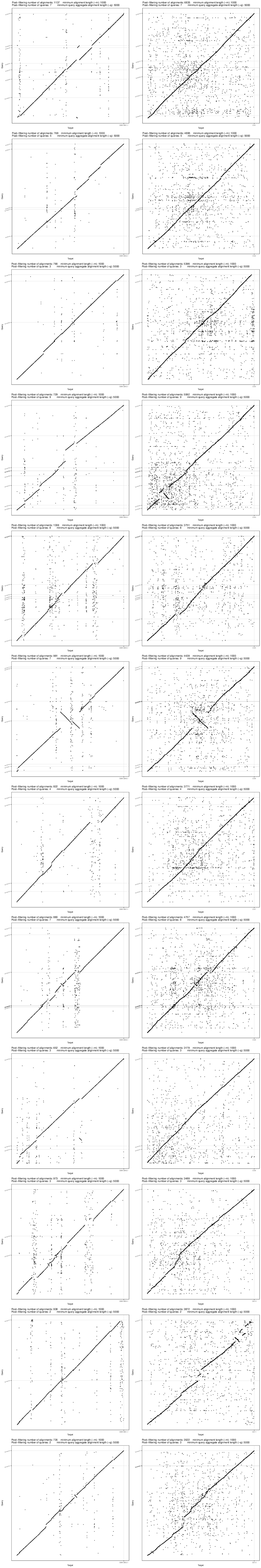

Supplement: S12 Fig — Chromosomes are shown in order 1 through 12. PacBio contigs from this study are on y-axis, Nanopore Carolina Gold Select [15] in first column and Oryza sativa var. indica in second column (ftp://ftp.gramene.org/pub/gramene/release-58/fasta/oryza_indica/dna/Oryza_indica.ASM465v1.dna_sm.toplevel.fa.gz). (PNG) [file pgen.1009389.s012.png]

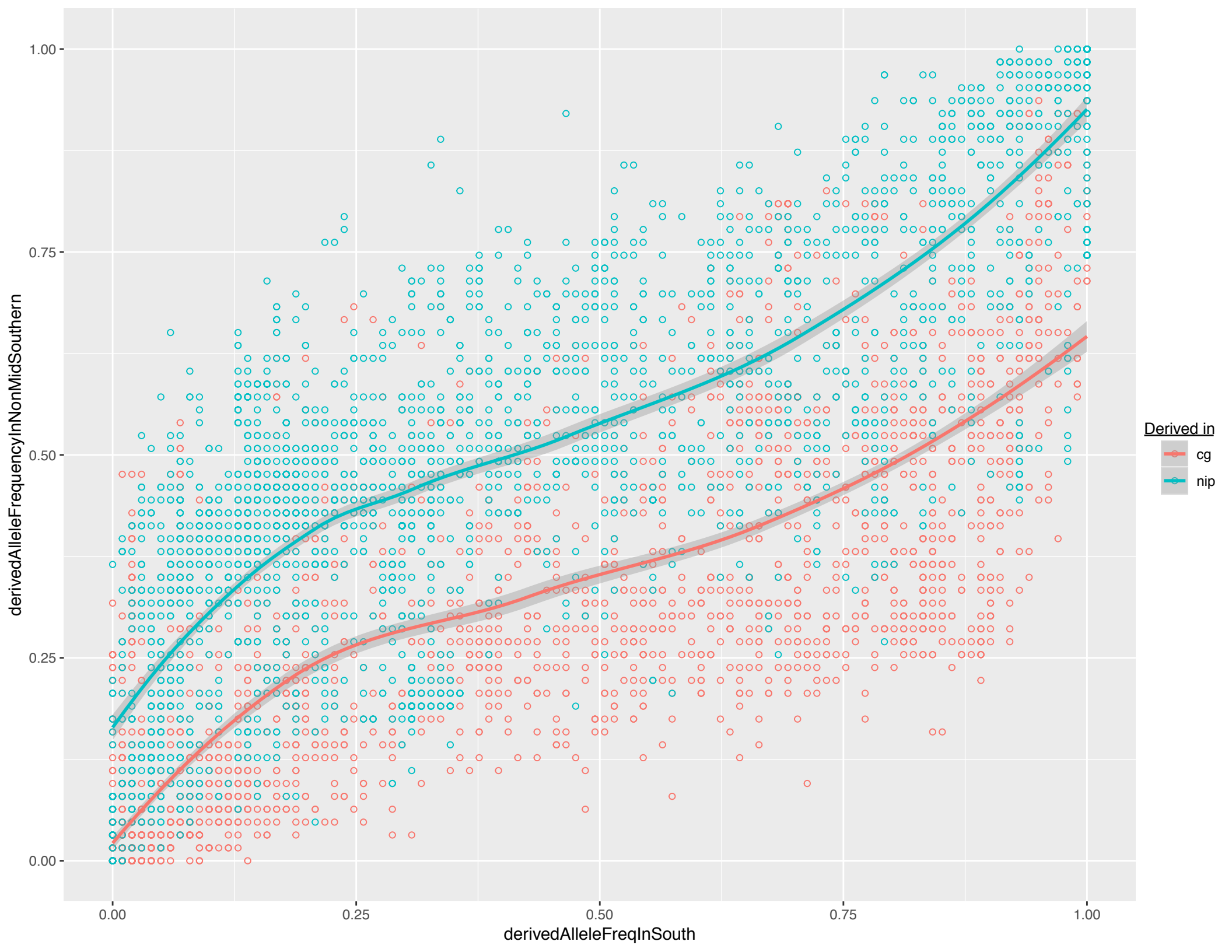

Supplement: S13 Fig — The reference containing the derived allele is indicated by color; line was fit using loess smoothing. (PDF) [file pgen.1009389.s013.pdf]

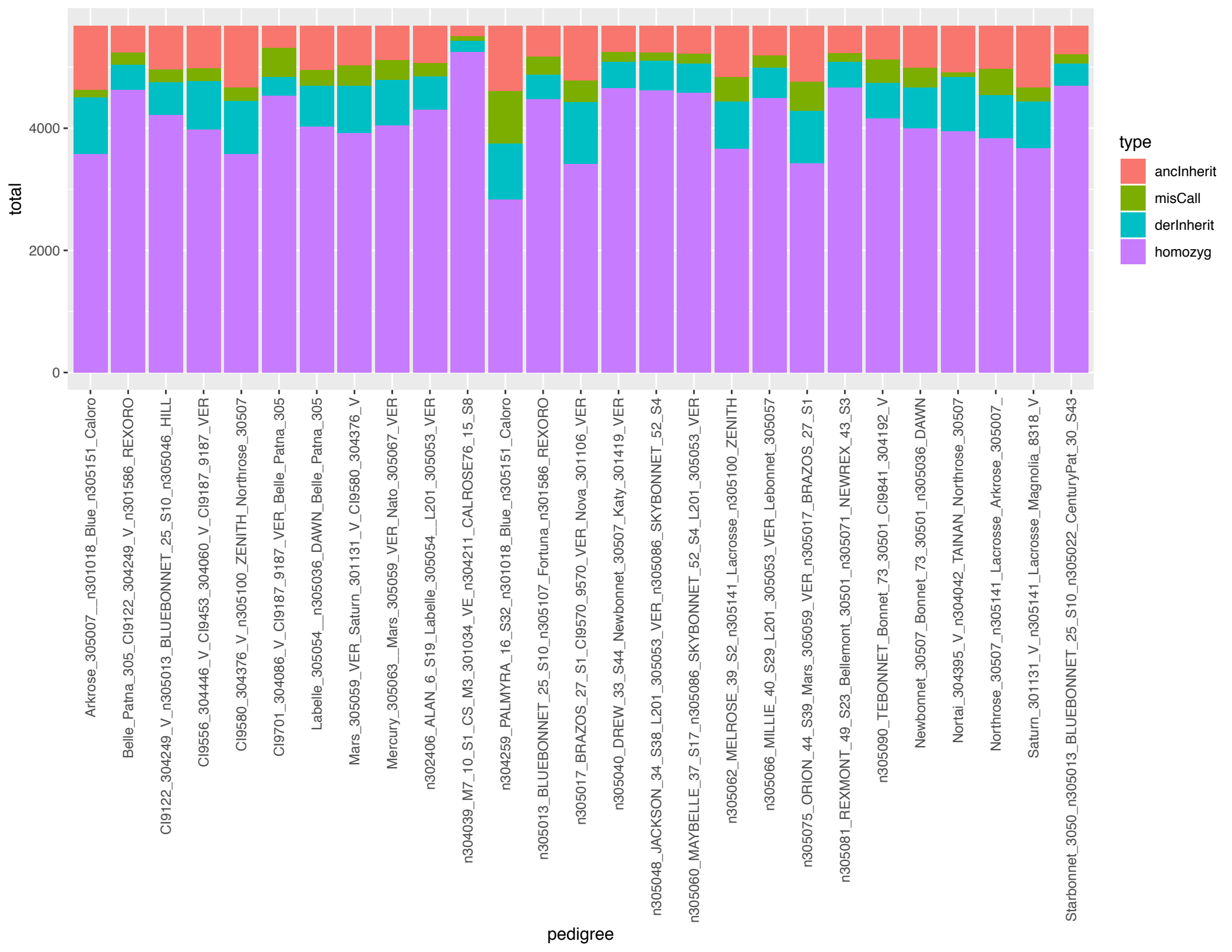

Supplement: S14 Fig — “ancInherit” and “derInherit” described cases in which the ancestral or derived allele was inherited, respectively. “misCall” describes cases in which the progeny allele is distinct from either parent allele. (PDF) [file pgen.1009389.s014.pdf]

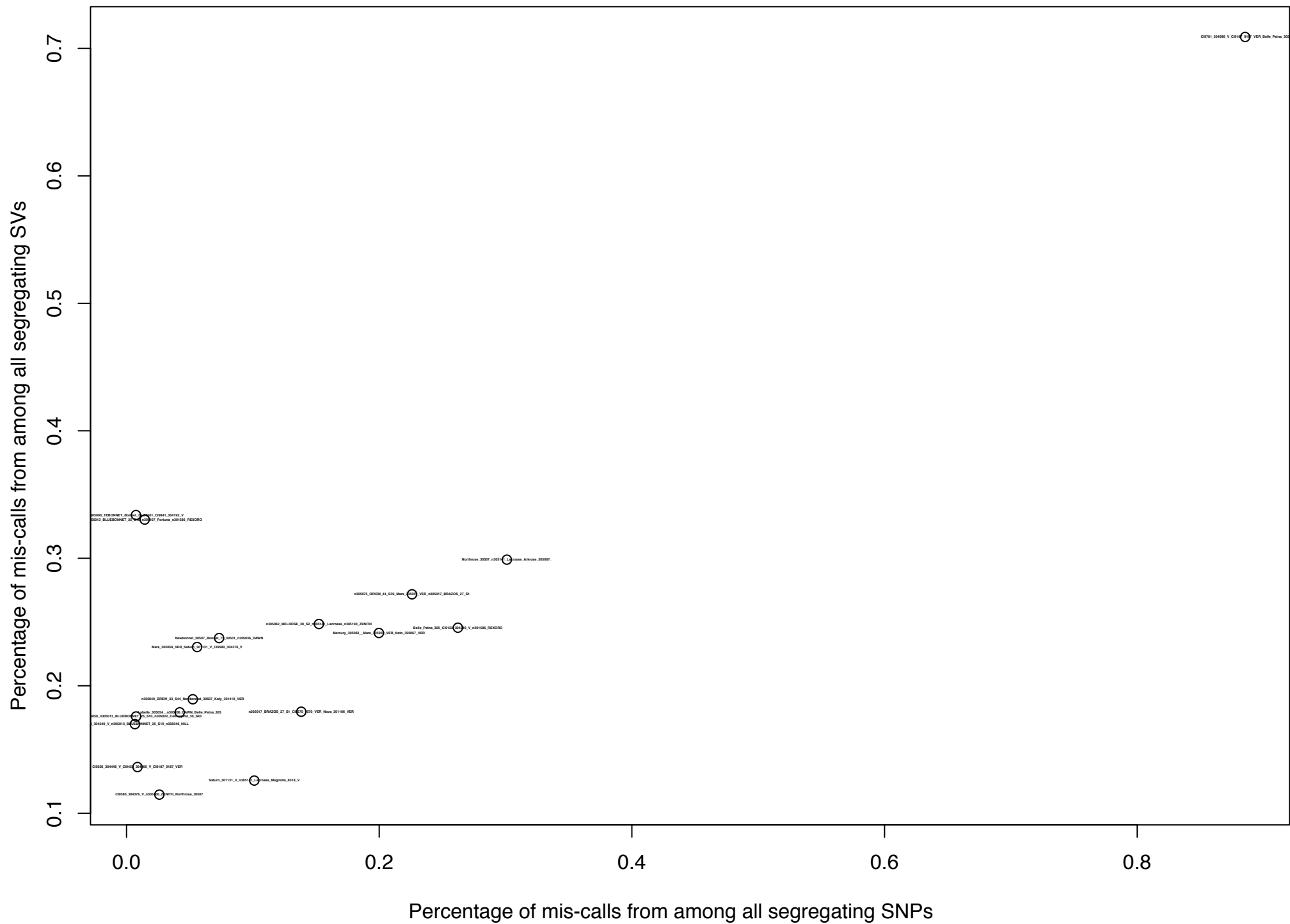

Supplement: S15 Fig — Note, 9 trios were removed from this comparison because they involved non-Midsouthern material and did not have complementary SNP data available. Axes represent the number of “misCalls”, as defined in S14 Fig, as a percentage of segregating variants; SVs on y-axis, SNPs on x-axis. (PDF) [file pgen.1009389.s015.pdf]

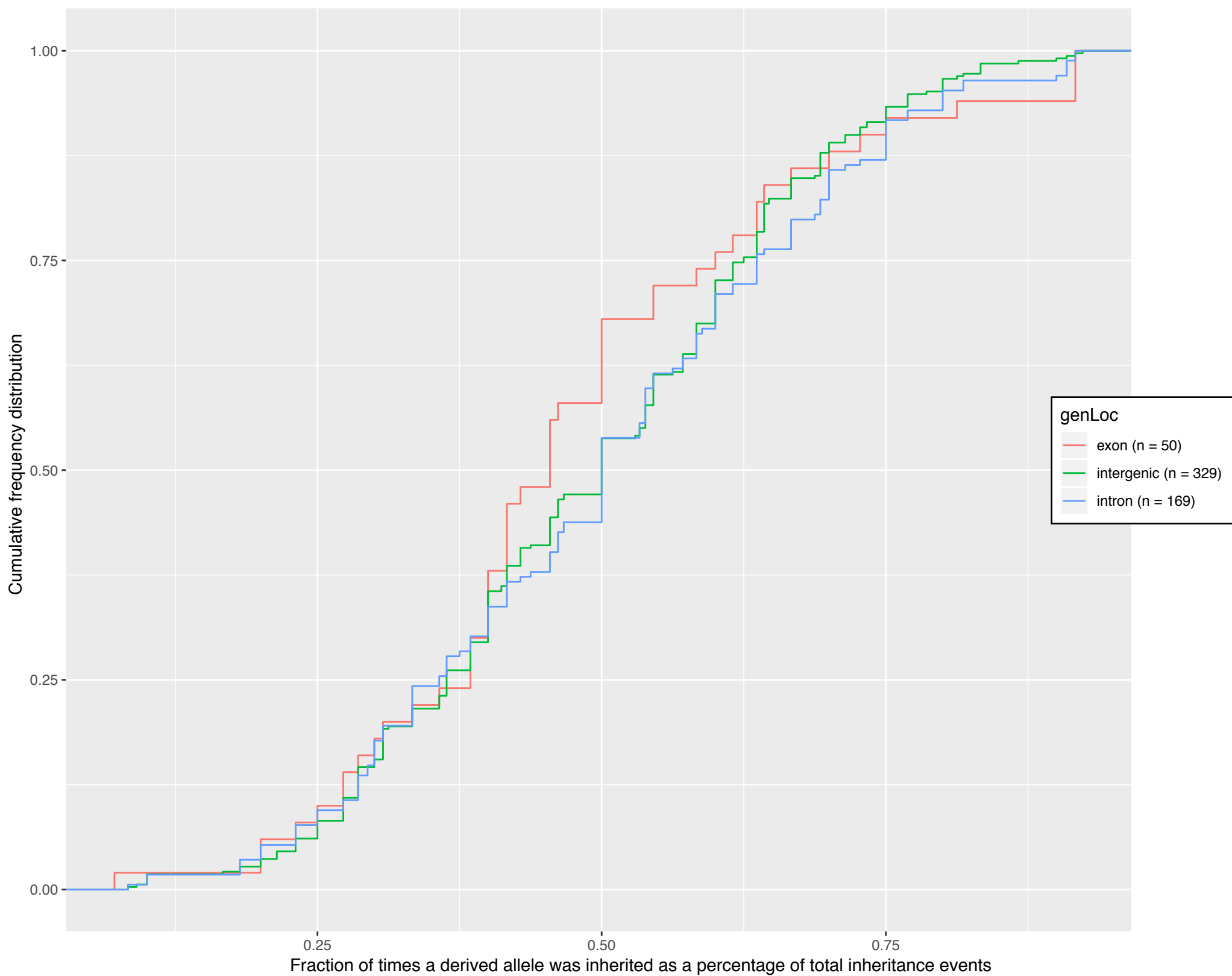

Supplement: S16 Fig — Any SV that had <10 trios segregating was removed. Among these, any SV for which either allele was never inherited–suggesting false parent call—was also removed. Final counts for each class are recorded in legend. (PDF) [file pgen.1009389.s016.pdf]
