# Supplementary material for: Gene disruption by structural mutations drives selection in US rice breeding over the last century
Source: PLoS Genet. 2021 Mar 18;17(3):e1009389. doi: 10.1371/journal.pgen.1009389 (PMC7971508; doi:10.1371/journal.pgen.1009389)
Supplement: S3 Fig — (PDF) [file pgen.1009389.s003.pdf]

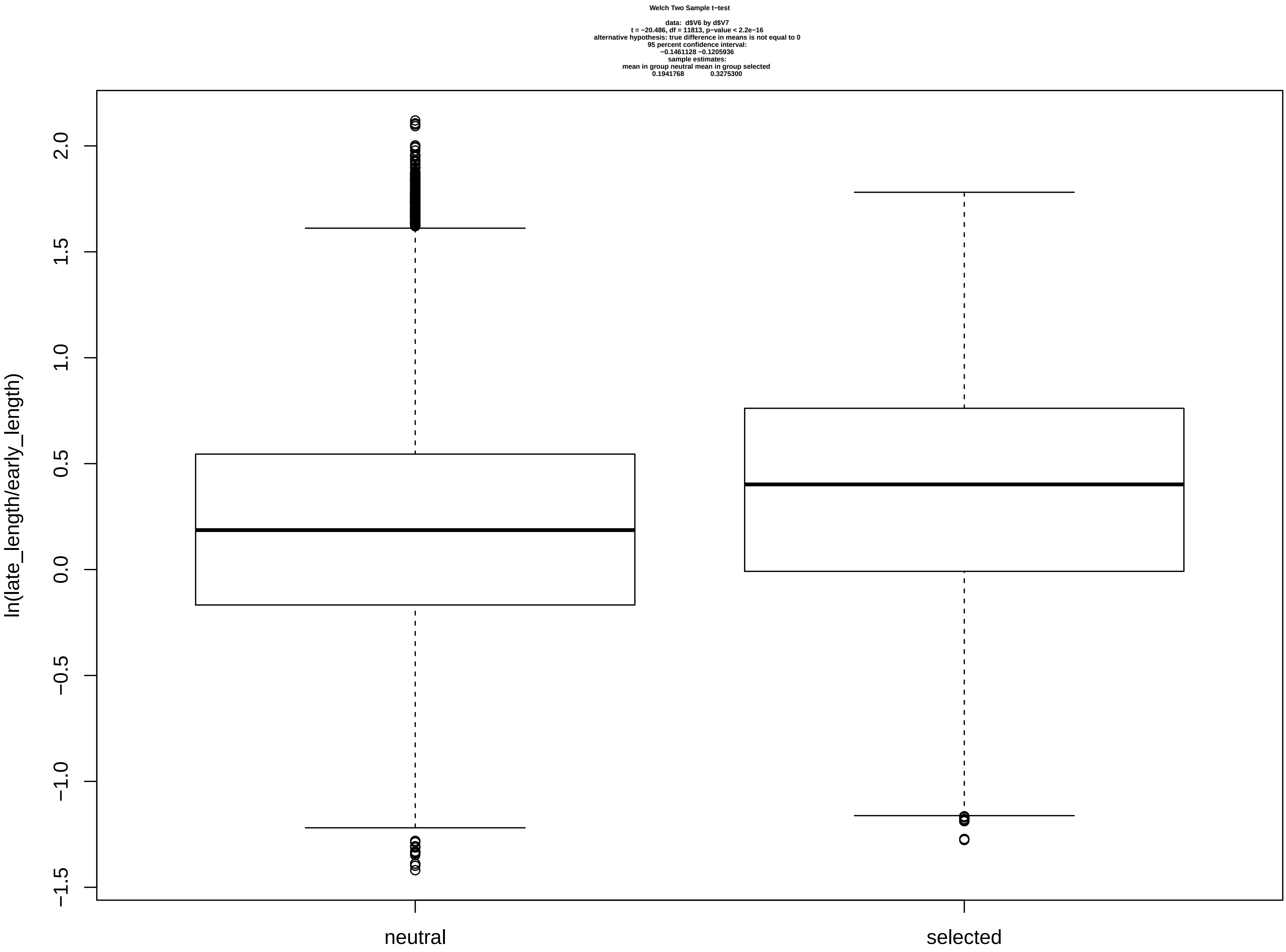
Welch Two Sample t−test
data: d$V6 by d$V7
t = −20.486, df = 11813, p−value < 2.2e−16
alternative hypothesis: true difference in means is not equal to 0
95 percent confidence interval:
−0.1461128 −0.1205936
sample estimates:
mean in group neutral mean in group selected
0.1941768 0.3275300
ln(late_length/early_length)
2.0
1.5
1.0
0.5
0.0
−0.5
−1.0
−1.5
neutral
selected
