# Supplementary material for: Gene disruption by structural mutations drives selection in US rice breeding over the last century
Source: PLoS Genet. 2021 Mar 18;17(3):e1009389. doi: 10.1371/journal.pgen.1009389 (PMC7971508; doi:10.1371/journal.pgen.1009389)
Supplement: S2 Table — (DOCX) [file pgen.1009389.s018.docx]

**Supplemental Table 2**: Core gene content for Carolina Gold assembly and Nipponbare reference

| **Result** | **Carolina Gold** | **IRGSP-1.0** |
| --- | --- | --- |
| Complete BUSCOs | 940 | 941 |
| Complete and single-copy BUSCOs | 912 | 917 |
| Complete and duplicated BUSCOs | 28 | 24 |
| Fragmented BUSCOs | 5 | 2 |
| Missing BUSCOs | 11 | 13 |
| Total BUSCO groups searched | 956 | 956 |
