# Supplementary material for: Gene disruption by structural mutations drives selection in US rice breeding over the last century
Source: PLoS Genet. 2021 Mar 18;17(3):e1009389. doi: 10.1371/journal.pgen.1009389 (PMC7971508; doi:10.1371/journal.pgen.1009389)
Supplement: S3 Table — (DOCX) [file pgen.1009389.s019.docx]

**Supplemental Table 3**: GO enrichment analysis using genes that have exonic SVs with a rate of < -0.00125 relative to derived allele (n = 67). Analysis used ShinyGO v0.61 with Oryza sativa Japonica Group as a background model.

| **Enrichment FDR** | **Genes in list** | **Total genes** | **Functional Category** |
| --- | --- | --- | --- |
| 3.0E-02 | 4 | 268 | Response to external biotic stimulus |
| 3.0E-02 | 4 | 268 | Response to other organism |
| 3.0E-02 | 4 | 237 | Defense response to other organism |
| 3.0E-02 | 4 | 275 | Response to biotic stimulus |
| 3.2E-02 | 5 | 498 | Multi-organism process |
| 3.6E-02 | 3 | 155 | Defense response to bacterium |
| 3.8E-02 | 3 | 168 | Response to bacterium |
| 5.0E-02 | 4 | 385 | Response to external stimulus |
