# Supplementary material for: Gene disruption by structural mutations drives selection in US rice breeding over the last century
Source: PLoS Genet. 2021 Mar 18;17(3):e1009389. doi: 10.1371/journal.pgen.1009389 (PMC7971508; doi:10.1371/journal.pgen.1009389)
Supplement: S4 Table — (DOCX) [file pgen.1009389.s020.docx]

**Supplemental Table 4**: GO enrichment analysis using genes that have exonic and intronic SVs with a rate of < -0.00125 relative to derived allele (n = 263). Analysis used ShinyGO v0.61 with Oryza sativa Japonica Group as a background model.

| **Enrichment FDR** | **Genes in list** | **Total genes** | **Functional Category** |
| --- | --- | --- | --- |
| 2.5E-02 | 4 | 41 | Rab protein signal transduction |
| 2.5E-02 | 6 | 155 | Defense response to bacterium |
| 2.5E-02 | 8 | 268 | Response to external biotic stimulus |
| 2.5E-02 | 8 | 268 | Response to other organism |
| 2.5E-02 | 5 | 94 | Supramolecular fiber organization |
| 2.5E-02 | 8 | 237 | Defense response to other organism |
| 2.5E-02 | 8 | 280 | Response to oxidative stress |
| 2.5E-02 | 4 | 60 | Ras protein signal transduction |
| 2.5E-02 | 8 | 275 | Response to biotic stimulus |
| 2.9E-02 | 4 | 66 | Small GTPase mediated signal transduction |
| 2.9E-02 | 6 | 168 | Response to bacterium |
| 4.9E-02 | 2 | 10 | Cortical microtubule organization |
| 4.9E-02 | 10 | 498 | Multi-organism process |
| 4.9E-02 | 23 | 1827 | Phosphorylation |
| 4.9E-02 | 4 | 85 | Defense response to oomycetes |
| 4.9E-02 | 4 | 85 | Response to oomycetes |
| 4.9E-02 | 9 | 425 | Defense response |
| 5.0E-02 | 2 | 12 | Cortical cytoskeleton organization |
