# Supplementary material for: Gene disruption by structural mutations drives selection in US rice breeding over the last century
Source: PLoS Genet. 2021 Mar 18;17(3):e1009389. doi: 10.1371/journal.pgen.1009389 (PMC7971508; doi:10.1371/journal.pgen.1009389)
Supplement: Attachment — Submitted filename: PGENETICS-D-20-01339_reviewer.docx [file pgen.1009389.s028.docx]

Remarks to the authors of “Gene disruption by structural mutations drives selection in US rice breeding over the last century”:

The study reports the detection of structural mutations and their population dynamics during modern US rice breeding. The purpose of the study is to detect structural mutations with >= 50 bp, and to characterize how these structural mutations were changed during modern US rice breeding. To address the questions, the study generated a de novo genome assembly of a *Tropical japonica* cv. Carolina Gold with PacBio long-read sequencing technology. Comparing the assembled genome sequence with Nipponbare reference genome with *O.glaberrima* as the outgroup yielded numerous of structural mutations. Tracing the variation of these structural mutations in 101 rice varieties bred at different eras in last century in the US showed they were more intensively selected than any other genomic variations during US rice breeding. To my opinion, recent rice genome study mainly focused on Asia landraces and varieties, the genetic architecture of US rice varieties remains unknown. This study could advance our understanding of genetic improvement during modern US rice breeding and provide a valuable resource for further comprehensive rice genome study. It is worthy of publication once following issues be addressed.

A. Major comments:
1. Rice recent functional genome studies have cloned many key genes for grain size and shape, eg, *GW2*, *GS3*, *GS5*, *qGL3*, *G1*, *GS6* and *qGW8*, the authors should evaluate the genotype change of these genes to elucidate the genetic basis underlying the grain shape change during US rice breeding, and evaluate the potential impact of structural mutations on these genes.
2. Structural mutations strongly correlated with the population structure. In the study, unevenly population structure with genetic introgression was observed during modern US rice breeding. The effect of population structure change and early genetic introgression on the purge of structural mutations should be evaluated before drawing the conclusion “Structural mutations	are being purged	from	the	germplasm”.
3. Recent rice genome studies have identified large number of key genes were selected during modern breeding (eg. Xie et al, Breeding signatures of rice improvement revealed by a genomic variation map from a large germplasm collection; Lin et al, Divergent selection and genetic introgression shape the genome landscape of heterosis in hybrid rice; Li et al, Analysis of genetic architecture and favorable allele usage of agronomic traits in a large collection of Chinese rice accessions). It is interesting that structural mutations were purged during US rice breeding. The functional effect of structural mutations on important agronomic important genes, or the relationship between structural mutations and recently reported selected genes would be helpful for explaining why structural mutations were selected during modern breeding.
B. Minor comments:
1. Lacking of reference or description to O.glaberrima genome in line 127.
2. As the genome assembly quality will affect the result of structural detection, the authors should provide a brief description about the quality of O.glaberrima genome.
3. Ambiguous words like “likely” or “roughly” should not be used to describe the results, eg at line176 and line 251, explicit number should be investigated across the manuscript.
4. More detailed information about the trait improvement during US rice breeding, eg. the quality, resistance and yield change, should be provided to help understanding the characteristic of US breeding improvement.
