# Supplementary material for: Gene disruption by structural mutations drives selection in US rice breeding over the last century
Source: PLoS Genet. 2021 Mar 18;17(3):e1009389. doi: 10.1371/journal.pgen.1009389 (PMC7971508; doi:10.1371/journal.pgen.1009389)
Supplement: Attachment — Submitted filename: ResonseToReviewers.docx [file pgen.1009389.s029.docx]

Response to reviewers (highlighted in yellow) for 'Gene disruption by structural mutations drives selection in US rice breeding over the last century' (PGENETICS-D-20-01339)

**Reviewer #1**: This paper describes the sequencing of Carolina Gold, a key rice variety in the US. The authors also do whole genome re-sequencing with 160+ varieties from the US breeding program. The authors use the generated sequence to examine the role of structural variants in the US breeding program, showing the pattern of SVs they observe and the shift over time.

This is a really nice paper that describes the dynamics of SVs during the breeding process. Normally papers that describe SVs in rice are not very compelling, but this one uses the data in an innovative way. It provides a glimpse of what happens in the course of breeding of a major crop, and is helped by the great amount of historical information (and seeds) of rice released in the US. In many ways, this could be a case study in historical genetics.

*Thank you for your encouragement. We sincerely appreciate that you have taken the time to understand our methodology and offered suggestions that have, we think, substantially strengthened the study.*

There are a few issues I think would help.

1. To what extent can they authors use the pedigree information and their ascertained SVs to even provide better resolution for the change in frequency over time; what SVs are generally lost?

*Great suggestion. We had discussed this possibility during manuscript development and assumed our number of segregating trios would be too low to be of sufficient statistical power. In response to this review, we revisited those numbers and interrogated the trios more fully. We were able to use 25 valid trios. The result, now described in the manuscript (lines 318-331 and lines 578-587), support conclusions based on the full year-of-release method, although as expected the pedigree approach involved far fewer comparisons.*

2. It would be good to provide some examples of strong change in SV frequency and what kind of genes or regions these are found in

*In response to this suggestion (and other reviewer comments), we have A) added more informative annotation information to the original indel supplemental table and B) examined GO enrichment for the most strongly declining derived alleles for exon and exon/intron disrupting SVs (new Supplemental Tables S3 and S4). We have briefly described these additions in the revised manuscript (lines 360-366).*

3. Can the authors compare their SVs in this dataset with the frequency found in the worldwide 3,000 genome collection? The US breeding population is essentially a relatively closed gene pool, so it would be interesting to see if SVs of high frequency in the worldwide japonica collection are low in the US and vice-versa.

*We were very excited to see the publication of an SV-specific analysis of the 3k data (*[*https://genome.cshlp.org/content/29/5/870.full.html*](https://genome.cshlp.org/content/29/5/870.full.html)*) during the preparation of this manuscript. Unfortunately, that data remains very problematic to evaluate. To be more specific, the material used is so diverse that a substantial fraction of the SVs are multi-allelic and so frequency assessment can be misleading: one snp within a large SV might create three alleles yet these alleles are not phylogenetically or phenotypically equidistant. We avoided this issue by focusing on the major insertion or deletions assuming that small internal mutations were insubstantial relative to the much larger event. We have contacted the authors to attempt to blend our results but the intermediate files needed to do so would require a major bioinformatic effort and many of the qualified authors have moved to other areas.*

*Still, because we sequenced many additional lines not in our Midsouth population (Figure 1) we can investigate the frequency of the SVs that we did describe within the context of that more diverse material. We have added a supplemental figure describing those results (****Supplemental Figure S13****) and a section in text (Lines 270-274). Results are generally as expected for CarGold versus Nipponbare derived alleles. Though allele frequencies are highly correlated in each set, there are examples of low frequency alleles in Midsouth material being relatively high in our more diverse sample.*

4. I think another Carolina Gold sequence was release last year (published also in PLos Genetics), but the authors do not refer to it at all. It would be good to refer to this paper and possibly make some comparison of the two genomes, given that the previously published paper used nanopore sequencing.

*We agree. The Nanopore assembly was released during preparation of this manuscript. We think that assembly was sufficient for the questions those authors were addressing, but we found that our contiguity metrics were much better and we were more confident in high-coverage PacBio quality than the Nanopore/Illumina strategy. Further analysis using Indica rice as a contrast bears this point out (see Figure S12). Again we feel both studies are sound, and we have attempted to incorporate their data as supporting information but to also point out our improvements (see updated lines 134-144). We welcome any additional editorial or reviewer suggestions on how to best acknowledge that manuscript.*

**Reviewer #2**: The study reports the detection of structural mutations and their population dynamics during modern US rice breeding. The purpose of the study is to detect structural mutations with >= 50 bp, and to characterize how these structural mutations were changed during modern US rice breeding. To address the questions, the study generated a de novo genome assembly of a Tropical japonica cv. Carolina Gold with PacBio long-read sequencing technology. Comparing the assembled genome sequence with Nipponbare reference genome with O.glaberrima as the outgroup yielded numerous of structural mutations. Tracing the variation of these structural mutations in 101 rice varieties bred at different eras in last century in the US showed they were more intensively selected than any other genomic variations during US rice breeding. To my opinion, recent rice genome study mainly focused on Asia landraces and varieties, the genetic architecture of US rice varieties remains unknown. This study could advance our understanding of genetic improvement during modern US rice breeding and provide a valuable resource for further comprehensive rice genome study. It is worthy of publication once following issues be addressed.

*Thank you, we also believe this study expands our understanding of US rice in particular and deleterious load in general. With regard to the US population, we think the app associated with this manuscript will be very helpful in case-by-case examination of the material (*[*https://gbru-ars.shinyapps.io/HaploStrata/*](https://gbru-ars.shinyapps.io/HaploStrata/)*). And thank you for your comments below; we think the changes they inspired have made the manuscript more exact and more widely applicable.*

Major comments:

Rice recent functional genome studies have cloned many key genes for grain size and shape, eg, GW2, GS3, GS5, qGL3, G1, GS6 and qGW8, the authors should evaluate the genotype change of these genes to elucidate the genetic basis underlying the grain shape change during US rice breeding, and evaluate the potential impact of structural mutations on these genes.

Recent rice genome studies have identified large number of key genes were selected during modern breeding (eg. Xie et al, Breeding signatures of rice improvement revealed by a genomic variation map from a large germplasm collection; Lin et al, Divergent selection and genetic introgression shape the genome landscape of heterosis in hybrid rice; Li et al, Analysis of genetic architecture and favorable allele usage of agronomic traits in a large collection of Chinese rice accessions). It is interesting that structural mutations were purged during US rice breeding. The functional effect of structural mutations on important agronomic important genes, or the relationship between structural mutations and recently reported selected genes would be helpful for explaining why structural mutations were selected during modern breeding.

*Note, we considered the two concerns directly above to be similar enough to answer with the following response:*

*In response to this concern, we have added genes/regions discussed in the grain size paper - Zhang et al, 2020, “Grain Size Selection Using Novel Functional Markers Targeting 14 Genes in Rice” - and the heterosis paper referred to above - Lin et al - to both our original indel table as well as our putatively selected regions table. The additional citations were not included: 1) Xie et al. was not used because it was focused on divergent selection in Indica subgroup and would not be applicable here and 2) Li et al. was not used because html links to the genome coordinates were no longer available. We should also note that the heterotic loci included from Lin et al are single-point positions; we expanded these +/- 1000 bp to check for adjacent SVs but we do not have a clear idea from that study as to what width we should be searching. Under these parameters, no grain size genes and 18 heterotic loci were overlapping with SVs used in our study, which is higher than the ~10 expected by chance, although rigorous significance is very difficult to assess given non-uniform distribution in the genome We have briefly describes these additions in revise manuscript (lines 360-366)*

*We agree that the overlap is interesting, but we should reiterate that our analysis is not exhaustive; we are only focused on alleles that could be conservatively defined as derived or ancestral, so some mutations in the above genes will be missed. This conservative approach allowed us to examine rates of change relative to an absolute reference: the progenitor state. Moreover, the trends we see in SV variation are hypothesized to be related with deleterious load, not specifically to QTL that are easily mapped. In effect, our approach is less specific than classical biparental mapping or GWAS but it is more sensitive. Large effect mutations - like those the reviewer cites - are more likely to overlap with results in the final section related to sweep windows. Indeed, sd1 - perhaps the most important agronomic gene - shows a distinctive signature. Yet, as our work indicates (and as many breeders who attempt to incorporate exotic haplotypes will attest), one qualitatively advantageous allele will often be swamped out by a deleterious haplotype.*

*We do think that, with the proper data from parents, the materials generated in Lin et al. would be incredibly useful in confirming many of the results of this manuscript.*

Structural mutations strongly correlated with the population structure. In the study, unevenly population structure with genetic introgression was observed during modern US rice breeding. The effect of population structure change and early genetic introgression on the purge of structural mutations should be evaluated before drawing the conclusion “Structural mutations are being purged from the germplasm”.

*We agree that population structure can be a powerful confounder when making inferences about one (or a few) genomic loci. Indeed, we have had to address this issue in prior work (Vaughn, et al. 2014, G3 and Vaughn, et al. 2016, G3). Our conclusions about exonic SVs are based on examining them as a class spread across the genome. Inter-chromosomal LD related to reproductive barriers is expected to manifest itself randomly across the genome. If our exonic SVs (or any category) had a biased concentration in the genome, such bias could potentially confound our results, but we do not observe it (Supplemental Figure S6).*

*In response to other reviewers, we also examined available pedigree trios in our data (see above). Since, trio analyses are independent of population structure, the complementary results support the assumption that structure is not confounding. A description of these results have been added to the manuscript (lines 318-331 and lines 578-587) along with supporting supplemental figures.*

*We also assess if early structure was a primary driver of our observations by checking rates using only varieties released after 1925. The results were highly correlated with those in the full set (see figure below, R2 = 0.88). In spite of the reduced sample set resulting from the cutoff, this similarity supports our conclusions regardless of the era of breeding. For the sake of clarity, we have not added this additional post-1925 analysis to the manuscript, but will do so if the reviewers/editors think it is warranted.*

Minor comments:

Lacking of reference or description to O.glaberrima genome in line 127.As the genome assembly quality will affect the result of structural detection, the authors should provide a brief description about the quality of O.glaberrima genome.

*We regret the omission of the correct citation, which has been added. The genome was assembled via a very different methodology, and we have added a brief description in the text (477-482). Notably, our SV ascertainment pipeline requires both macro- and micro-colinearity and so is robust to variability in assembly methodology and variation in quality, assuming errors are not convergent. To that end, it is actually advantageous that all genomes we used for the ascertainment step were generated via very diverse methods as it reduces the likelihood of these convergent errors.*

Ambiguous words like “likely” or “roughly” should not be used to describe the results, eg at line176 and line 251, explicit number should be investigated across the manuscript.

*We very much appreciate catching these ambiguities and have attempted to delete or clarify where needed. In one case (line 293), we left the “roughly” ambiguity because the order is not perfect, but we are happy to change if a better phrasing can be suggested. “Likely” was much more difficult to weed out, although we have reevaluated every instance to determine if we could be more decisive on the point in question.*

More detailed information about the trait improvement during US rice breeding, eg. the quality, resistance and yield change, should be provided to help understanding the characteristic of US breeding improvement.

We can added Introductory text and citations that we hope will address this concern (lines 91-103).

**Additional note**: For continuity and simplicity, main figures have been retained in the manuscript. Upon acceptance, we will be happy to process them through PACE system and apply any additional formatting request.
